# Quantifying metabolic decrement in brain FDG-PET of a complex frontotemporal dementia case

**DOI:** 10.1590/1980-5764-DN-2024-0277

**Published:** 2025-10-24

**Authors:** Raphael de Luca e Tuma, Artur Martins Coutinho, Camila de Godoi Carneiro, Luciana Cassimiro, Lucas Assis Santos de Souza, Carlos Alberto Buchpiguel, Leonel T. Takada, Sonia Maria Dozzi Brucki

**Affiliations:** 1 Universidade de São Paulo, Faculdade de Medicina, Departamento de Neurologia, São Paulo SP, Brazil.; 2 Universidade de São Paulo, Faculdade de Medicina, Departamento de Radiologia e Oncologia, São Paulo SP, Brazil.

**Keywords:** Frontotemporal Dementia, Positron-Emission Tomography, Fluorodeoxyglucose F18, Demência Frontotemporal, Tomografia por Emissão de Pósitrons, Fluordesoxiglucose F18

## Abstract

Diagnosis of behavioral variant frontotemporal dementia (bvFTD) in its initial stages can be supported by brain [18F] fluorodeoxyglucose-positron emission tomography (FDG-PET). The routine use of semi-quantitative analyses in FDG-PET is formally recommended. However, the longitudinal changes observed in sequential FDG-PETs scans throughout neurodegenerative diseases remain insufficiently understood. In this case, a bvFTD phenocopy remained clinically stable for nine years before exhibiting functional decline consistent with bvFTD. Hypometabolism compatible with bvFTD was observed only on the third FDG-PET, performed six years after the first. Retrospective analysis revealed that the second FDG-PET had already shown a decline in metabolism in areas typically affected by bvFTD compared to the first scan. During the period of clinical stability, individual exams were insufficient to confirm the diagnosis. However, the metabolic decrease between the first and second scans may have indicated subclinical progression. The decline in semi-quantitative FDG-PET scores could represent a useful diagnostic tool in bvFTD.

## RESUMO

O diagnóstico da variante comportamental da demência frontotemporal (DFTvc) em seus estágios iniciais pode ser auxiliado pela tomografia por emissão de pósitrons com [18F]fluordeoxiglicose (PET-FDG). O uso rotineiro de análises semiquantitativas em exames de PET-FDG é formalmente recomendado. Entretanto, as mudanças longitudinais observadas em exames PET-FDGs repetidos ao longo da evolução de doenças neurodegenerativas ainda são pouco compreendidas. Neste caso, uma fenocópia de DFTvc permaneceu clinicamente estável por nove anos antes de apresentar um declínio funcional compatível com DFTvc. O hipometabolismo típico da DFTvc foi identificado apenas na terceira PET-FDG, realizada seis anos após a primeira. Uma análise retrospectiva revelou que a segunda PET-FDG já demonstrava redução do metabolismo em áreas geralmente afetadas pela DFTvc, quando comparado ao primeiro exame. Durante o período de estabilidade clínica, exames individuais não foram suficientes para confirmar o diagnóstico. No entanto, a redução metabólica entre o primeiro e o segundo exame poderia indicar uma progressão subclínica subjacente. O decremento metabólico semiquantitativo pode ser uma ferramenta diagnóstica útil na DFTvc.

## INTRODUCTION

 Diagnosis of behavioral variant frontotemporal dementia (bvFTD) in prodromal or mild stages poses numerous challenges. Behavioral disturbances are an early manifestation of the disease, and neuropsychiatric symptoms such as apathy and disinhibition can precede executive dysfunction and other forms of cognitive impairment^1,2^, which are not mandatory to establish a diagnosis of possible or probable bvFTD^3^. Although other neurodegenerative diseases can present with behavioral symptoms^4^, patients with prolonged periods of stability without cognitive or functional impairment present a distinct set of differential diagnoses. Specifically, they can be difficult to distinguish from primarily psychiatric disorders^5^, which may or may not have been present throughout the patient’s life, or from an entity referred to as bvFTD phenocopy. Phenocopies typically present behavioral symptoms that may fulfill bvFTD diagnostic criteria but do not progress to dementia and do not display conclusive neurodegenerative findings on brain magnetic resonance imaging (MRI) or [^18^F]fluorodeoxyglucose-positron emission tomography (FDGPET)^6,7^. These distinctions carry important prognostic implications for both the patient and their family. 

 Patients with bvFTD can exhibit changes in the anterior frontal, temporal, and cingulate cortices, which may appear as atrophy on MRI^8^ or hypometabolism on FDG-PET^9^. Brain FDG-PET has been shown to detect functional abnormalities typical of bvFTD in some cases before MRI reveals characteristic structural changes^10^. In addition to simple visual analysis of FDG-PET, tools for semi-quantitative assessment can further support the diagnosis of neurodegenerative diseases, increasing both specificity and diagnostic confidence^11^. However, even with the use of FDG-PET and semi-quantitative analysis, some patients may not reach a definitive diagnosis. Repeating FDG-PET in unresolved cases can lead to a change in the primary diagnosis and enhance diagnostic confidence^12^. 

## CASE REPORT

**Figure 1:**
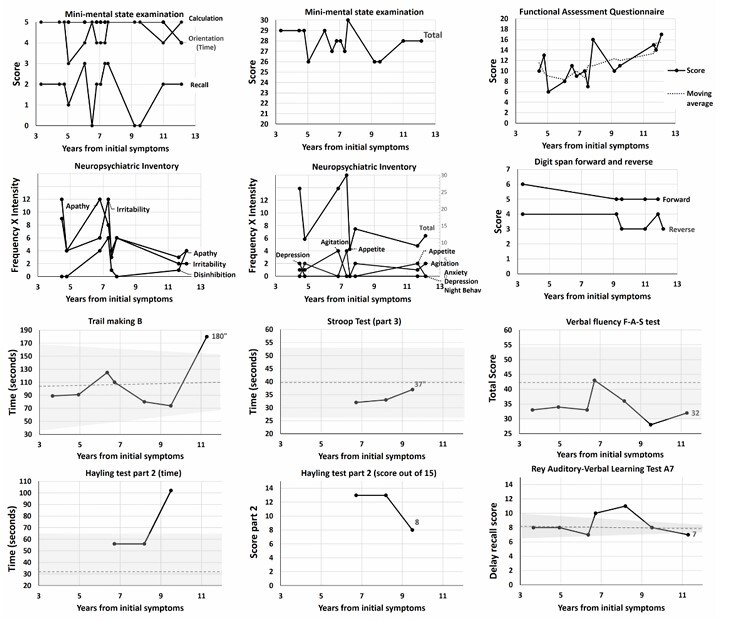
Clinical test scores from the initial evaluation, conducted three years after symptom onset, through to 11 years from symptom onset. Consistent reports of cognitive, behavioral, and functional decline occurred between eight and nine years after initial symptoms. Functional assessment questionnaire scores are presented alongside a moving average calculated from the previous, current, and subsequent scores to reduce variability. Dashed lines indicate average scores for normal controls, and greyed-out bands represent ±1 standard deviation relative to age, where applicable^13-16^.

 A 68-year-old man presented with a three-year history of memory loss, which began shortly after retiring as a systems analyst. He described difficulty recalling recent events as well as navigating familiar places. His wife reported that he had become repetitive and had forgotten the password to his bank account. Despite these difficulties, the patient was able to perform his instrumental activities of daily living independently. During the same period, he also displayed excessive irritability, impatience, and marked apathy, unlike his previous self. Family members attributed his cognitive difficulties to these behavioral symptoms, which often led to a quick loss of interest and a short attention span. He had 20 years of formal education, no prior psychiatric history, and no family history of dementia. The patient achieved normal scores in both basic cognitive screening and neuropsychological tests (Figure 1)^13-16^, with no evidence of memory or executive functional impairment. Physical examination revealed no signs of parkinsonism, eye movement abnormalities, or frontal disinhibition. Brain MRI was unremarkable, and visual analysis of FDG-PET by a nuclear medicine physician was described as normal. 

**Figure 2:**
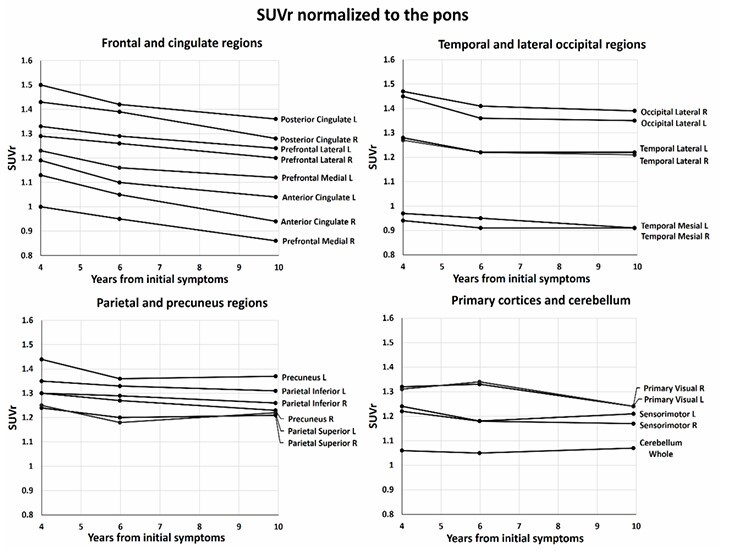
Standardized uptake value ratios (SUVr) from FDG-PET scans, normalized to the pons for the initial, second, and final scans, performed approximately four, six, and ten years after the onset of reported symptoms, respectively. An SUVr of 1 indicates metabolic activity equal to that of the pons. The interval between the first and second scans was 755 days, and between the second and final scans, 1,499 days. Quantification was performed using the CortexID Suite® software (GE Healthcare). Abbreviations: R, right; L, left. Note: The software predetermines all regions.

**Figure 3:**
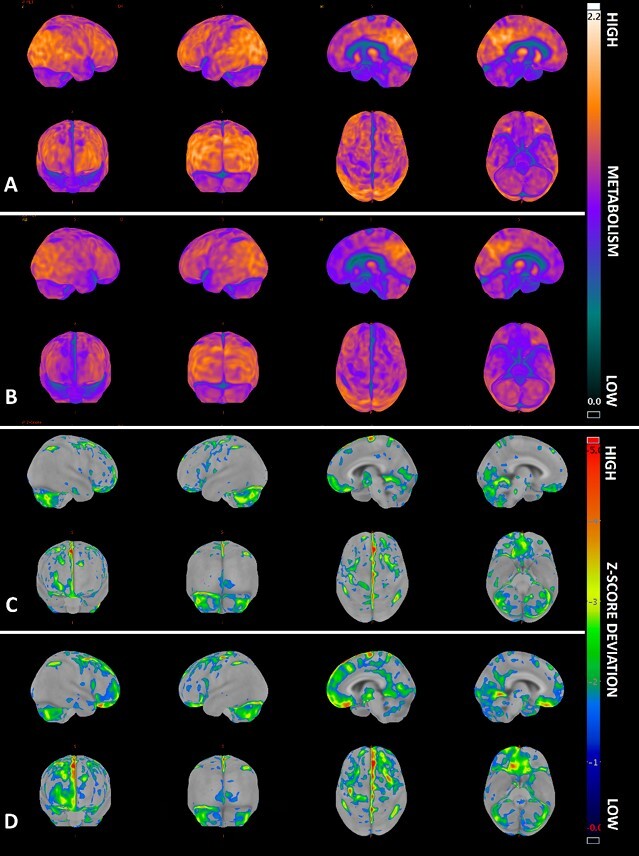
3D-Stereotactic Surface Projection (3D-SSP, CortexID Suite® software) of the patient’s first FDG-PET (A&C) and final FDG-PET (B&D), performed six years later. A and B) 3D-SSP maps of brain metabolism relative to (normalized by) the pons. Blue to purple indicate comparatively low metabolism, while orange indicates comparatively high metabolism. C and D) Z-score 3D-SSP maps comparing the patient to an age-matched healthy control group. Grey represents minimal or no deviation; blue to green, mild deviations; and yellow to red, greater deviations. The final FDG-PET demonstrates hypometabolism in the medial frontal cortex, orbitofrontal cortex, and anterior cingulate gyri, more pronounced in the right hemisphere. From top to bottom and left to right, images displayed are: right lateral, left lateral, right medial, left medial, anterior, posterior, superior, and inferior views.

 An initial diagnosis of major depressive disorder was established, although a neurodegenerative disease was not ruled out pending long-term follow-up. Escitalopram dosage was increased, which partially improved irritability, though the patient continued to exhibit apathy, indifference, and self-neglect. Over the following six years, family members reported some periods of subjective worsening without objective progression in functional impairment, although symptoms were largely described as stable. Some tests showed a slight decline from baseline but remained mostly within normal parameters, with some fluctuating scores (Figure 1). A repeat FDG-PET two years after the first remained normal on visual analysis. Nine years after the initial symptoms, family members reported consistent cognitive and behavioral deterioration, including excessive spending, inappropriate comments, and reckless and aggressive driving. The Frontal Assessment Battery (FAB) score was considered normal at 15/18, but the INECO Frontal Screening (IFS) test revealed impairments in specific subtests (Table 1)^17-19^. Executive function showed a significant decline later in the disease course (Figure 1). A final FDG-PET, performed six years after the first and nearly ten years after symptom onset, revealed findings not presented in the previous scans. Visual and semi-quantitative analyses showed mild to moderate hypometabolism in prefrontal regions, notably the medial frontal cortex, orbitofrontal cortex, and anterior cingulate gyri, more prominent in the right hemisphere. Glucose uptake was also asymmetrical in the anterior temporal lobes, with right <left (Figures 2 and 3). 

**Table 1:** INECO frontal screening test and Ekman faces test, administered later during follow-up. Averages and standard deviations for normal controls in the INECO test are shown for the Brazilian population, and for the original population in the facial emotion recognition test (Ekman pictures), Mini-SEA version^17-19^.

Prefrontal testing				
Years of symptoms	~11.3 years	~11.8 years	~12.2 years	Normal
INECO frontal screening test total	17/30	19/30	18/30	21.97±3.68
Motor programming	3/3	3/3	3/3	2.73±0.59
Conflicting instructions	3/3	3/3	3/3	2.80±0.41
Go-no go	3/3	2/3	2/3	2.60±0.41
Digit backwards	3/6	3/6	1/6	3.27±1.49
Verbal working memory	2/2	1/2	2/2	1.87±0.35
Spatial working memory	2/4	1/4	3/4	2.13±0.74
Abstraction capacity	0/3	0/3	0/3	2.57±0.62
Verbal inhibitory control	1/6	6/6	4/6	4.13±1.51
Facial emotion recognition test (Mini-SEA)	9.0/15		10.7/15	12.55±1.15

 A final diagnosis of probable bvFTD was established based on clinical signs of disinhibition — manifested as socially inappropriate behavior and poor decision-making — alongside apathy and a neuropsychological profile revealing executive function deficits with relative preservation of episodic memory and visuospatial skills. Functional decline was confirmed by functional activities questionnaire scores (Figure 1). Finally, FDG-PET findings were consistent with bvFTD. 

 All three FDG-PET scans were acquired using a GE Discovery 710 PET/CT scanner (GE Healthcare, Milwaukee, WI, USA). Quantitative 3D-SSP analyses were performed using the commercial software CortexID Suite® (GE Healthcare). Glucose uptake was measured by standardized uptake value ratio (SUVr) in each pre-defined key region, normalized to both average uptake in the pons and to global cerebral uptake. Figure 3 presents the 3D-SSP model constructed from the first and final FDG-PETs. The software also provided a table with average SUVr and Z-score for predefined reference regions, including the pons (Table 2). All regions showed negative Z-scores across all three exams when normalized to the pons. In the first FDG-PET, the lowest Z-score was in the right medial prefrontal cortex, followed by both primary visual cortices and then the primary sensorimotor cortices. In the final FDG-PET, the lowest Z-score remained in the right medial prefrontal cortex, and the same pattern involving primary cortices persisted. Normalization to global mean cortical uptake yielded similar results. However, when assessing the absolute difference in SUVrs between the first and final exams, the greatest declines were observed in the bilateral anterior cingulate and posterior cingulate regions, similar to those in the medial prefrontal lobes, with changes in the primary cortices being comparatively minor. When SUVr values were normalized to global cortical uptake, variations follow a similar pattern, albeit with smaller differences. 

**Table 2:** Standardized uptake value ratios (SUVr) for regions predefined by CortexID Suite® (GE Healthcare) software, normalized to the pons, presented along with Z-score comparisons to age-matched healthy controls. An SUVr of 1 indicates an average uptake equal to the average uptake of the pons. A Z-score of 0 denotes the patient’s SUVr matches the average of the healthy control group. A Z-score of -1 indicates one standard deviation below average, and a Z-score of +1 indicates one standard deviation above the average.

SUVr and z-scores normalized to the pons						
Region	FDG-PET 1 (4 years)		FDG-PET 2 (6 years)		FDG-PET 3 (10 years)	
	SUVr	Z-score	SUVr	Z-score	SUVr	Z-score
Prefrontal Lateral (R)	1.29	-2.01	1.26	-2.27	1.2	-2.35
Prefrontal Lateral (L)	1.33	-1.54	1.29	-1.84	1.24	-1.94
Prefrontal Medial (R)	1	-3.75	0.95	-4.19	0.86	-4.21
Prefrontal Medial (L)	1.23	-1.52	1.16	-2.24	1.12	-2.13
Sensorimotor (R)	1.22	-2.25	1.18	-2.58	1.17	-2.84
Sensorimotor (L)	1.24	-2	1.18	-2.51	1.21	-2.28
Anterior Cingulate (R)	1.13	-1.01	1.05	-1.62	0.94	-1.81
Anterior Cingulate (L)	1.19	-0.49	1.1	-1.23	1.04	-1.29
Posterior Cingulate (R)	1.43	-1.45	1.39	-1.73	1.28	-2.08
Posterior Cingulate (L)	1.5	-0.93	1.42	-1.52	1.36	-1.61
Precuneus (R)	1.3	-2.21	1.27	-2.42	1.23	-3.34
Precuneus (L)	1.44	-1.28	1.36	-1.8	1.37	-1.96
Parietal Superior (R)	1.24	-1.34	1.2	-1.67	1.21	-1.71
Parietal Superior (L)	1.25	-1.18	1.18	-1.68	1.22	-1.59
Parietal Inferior (R)	1.3	-1.39	1.29	-1.49	1.26	-1.56
Parietal Inferior (L)	1.35	-0.84	1.33	-1.05	1.31	-1.01
Occipital Lateral (R)	1.47	-0.54	1.41	-1.05	1.39	-0.96
Occipital Lateral (L)	1.45	-0.65	1.36	-1.33	1.35	-1.3
Primary Visual (R)	1.32	-2.27	1.33	-2.2	1.24	-2.31
Primary Visual (L)	1.31	-2.29	1.34	-2.1	1.24	-2.34
Temporal Lateral (R)	1.27	-0.67	1.22	-1.12	1.21	-1.04
Temporal Lateral (L)	1.28	-0.35	1.22	-1.03	1.22	-0.88
Temporal Mesial (R)	0.94	-1.58	0.91	-2	0.91	-1.88
Temporal Mesial (L)	0.97	-0.92	0.95	-1.26	0.91	-2.05
Cerebellum Whole	1.06	-2.79	1.05	-2.83	1.07	-2.31
Pons	1	0	1	0	1	0

Abbreviations: SUVr, standardized uptake value ratio; FDG-PET, fluorodeoxyglucose-positron emission tomography; R, right; L, left.

## DISCUSSION

 This case exemplifies the difficulty in categorizing patients with prolonged clinical stability and normal imaging findings, as well as the challenges in documenting progression in cases with predominantly behavioral symptoms. However, the semi-quantitative values of the second FDG-PET, compared to the first, were notable. Hypometabolism compatible with bvFTD did not become overt until the third exam, but individual regional values in the semi-quantitative analyses clearly showed a decline even during the initial period (Figure 2). This was particularly evident in the anterior cingulate cortex and, to a lesser extent, the association cortices between the first and second scans. Although the individual SUVrs did not cross thresholds indicative of underlying pathology, the variation or decrement itself was the only sign of progression in an otherwise stable patient. Whether this variation could serve as a specific biomarker has yet to be fully explored. This finding, in conjunction with the reported symptoms, could have supported the diagnosis of bvFTD prior to the third exam. 

### Progression in glucose hypometabolism as a diagnostic finding

 The first FDG-PET (Figure 3), along with the second, appeared to show deviations from normalcy, although these alterations remained non-specific. Psychiatric disorders can also present with metabolic abnormalities^20^, adding to the complexity of interpreting imaging studies with less pronounced changes. Individual regional Z-scores showed deviations in areas not typically associated with bvFTD or other neurodegenerative diseases, particularly the primary sensorimotor and visual cortices — a finding of uncertain clinical significance. Results may be affected by warping when translating the patient’s voxels to the predetermined mapped points due to anatomical differences between individuals, as well as by artifacts from focal lesions or atrophy. The normalization process^21^ can also generate relatively small and non-specific areas of hypometabolism in regions without pathological changes^22^. These caveats underscore both the importance of a review by a trained professional and the limitations of isolated scans in the early stages of the disease. 

 Nonetheless, regions typically associated with bvFTD not only appeared to have relatively lower metabolism at clinical presentation but also showed a more significant and consistent decline over time, particularly in the right anterior cingulate. Primary cortices and the cerebellum seemed relatively stable, though changes in the primary visual cortex remain unexplained, as they are more characteristic of Lewy Body Dementia^23^. Other regions related to association cortices had higher values and smaller decrements, except for the posterior cingulate bilaterally — a finding more specific to Alzheimer Disease (AD), but one that has also been described in bvFTD^9,24^. The degree of decrement between the first two FDG-PETs, when the patient was largely stable, appears almost as pronounced as that between the second and final exams. 

 The 3D-SSP map (Figure 3) presents a more specific pattern than individual regional scores. This may be due to hypometabolic areas affecting smaller surfaces within the predefined regions available in the software. These limitations are further compounded when the predefined regions are based on AD patterns while attempting to analyze a case of bvFTD. Visually, the 3D-SPP map showed greater hypometabolism in orbitofrontal regions and asymmetry in the anterior temporal lobes. The software does not automatically generate these specific regions, instead quantifying lateral and medial frontal cortices and lateral and medial temporal cortices, potentially diluting the values of affected areas with those of more stable regions. 

 Although various software programs and PET scanners are available, the normalization process combined with automated demarcation of anatomical regions appears to significantly improve the reproducibility of findings. A commercially available software package was used that does not require in-house protocols or user-generated regions of interest, allowing for adequate comparison of semi-quantitative values across studies, especially for future meta-analyses. As normalization generates a ratio of values within the same scan, external factors, such as differences in imaging protocols, are largely mitigated, since both values in the ratio are similarly affected. 

 Patterns of hypometabolism have been well described in cross-sectional studies, and longitudinal changes in FDG-PET have also been reported^25^. However, the rate at which hypometabolism progresses in neurodegenerative diseases compared to healthy controls is not fully understood. It is reasonable to expect relative stability in psychiatric disorders, in contrast to neurodegenerative conditions, and that metabolic decline would precede the emergence of specific patterns. Furthermore, patient-specific artifacts would likely remain consistent across repeated scans. Regardless, when specialists in cognitive and behavioral neurology interpret molecular imaging studies, they should be mindful of the existence of semi-quantitative assessment for longitudinal analysis. 

### Clinical diagnosis and evaluation

 The patient initially received a psychiatric diagnosis of depression prior to the diagnosis of bvFTD, a common occurrence^26^. This may be explained by a distinct prodromal stage characterized by psychiatric symptoms or by the fact that early-stage bvFTD can mimic depression^27^. There was an initial positive response to antidepressants and subsequent worsening may reflect progression of the underlying neurodegenerative process. This could account for some later test scores resembling baseline scores, with an interim period of improvement more accurately representing the patient’s functional baseline. In such cases, a peak-to-trough comparison could be more useful than a start-to-finish comparison. A prodromal psychiatric phase would also temporally overlap with a predominantly behavioral neurodegenerative disorder, resulting in a longer symptomatic period. 

 The initial presentation could alternatively be labeled as mild behavioral impairment (MBI), an entity proposed to identify prodromal bvFTD cases presenting with neuropsychiatric symptoms prior to the development of cognitive impairment and, eventually, dementia^1^. However, the patient had been diagnosed with major depressive disorder, which precludes the diagnosis of MBI^28^. Classification as a bvFTD phenocopy appears more pragmatic, given the extended period of clinical stability prior to progression, normal imaging findings, and the differential diagnosis with a primary psychiatric disorder. 

 As with other neurodegenerative diseases, diagnosis during preclinical stages may be a key prerequisite for future clinical trials. FDG-PET had already been shown to improve diagnostic accuracy in such stages^29^, and it may also be used to further stratify this population. Once a patient reaches overt stages of dementia, numerous scales and tests can be employed to gauge disease progression and consistently define sub-stages. In contrast, classification in preclinical and prodromal stages remains largely binary and lacks comparable granularity, despite the gradual nature of neuropathological progression^30^. Longitudinal semi-quantitative FDG-PET could assist in predicting proximity to clinical conversion and in assigning stages within the prodromal phase. 

 In conclusion, small changes in brain metabolism may remain imperceptible on visual inspection of FDG-PET scans yet still reflect progression of synaptic dysfunction. These subtle decrements, detectable through semi-quantification and considered alongside clinical evaluation, may support the diagnosis of prodromal bvFTD or offer insights into preclinical stages. Clinicians are justified in requesting a second FDG-PET scan when the first is inconclusive and should be aware of the potential for longitudinal changes in semi-quantitative FDG-PET values to demonstrate progressive decline in affected regions before reaching the threshold of clinical detectability. 

## Data Availability

The datasets generated and/or analyzed during the current study are available from the corresponding author upon reasonable request.
